# Replacement of microsatellite markers by imputed medium-density SNP arrays for parentage control in German warmblood horses

**DOI:** 10.1007/s13353-022-00725-9

**Published:** 2022-09-29

**Authors:** Wietje Nolte, Hatem Alkhoder, Mirell Wobbe, Kathrin F. Stock, Ernst Kalm, Sarah Vosgerau, Nina Krattenmacher, Georg Thaller, Jens Tetens, Christa Kühn

**Affiliations:** 1 Research Institute for Farm Animal Biology, Institute of Genome Biology, 18196 Dummerstorf, Germany; 2 Saxon State Office for Environment, Agriculture and Geology, 01468 Moritzburg, Germany; 3 IT Solutions for Animal Production, 27283 Verden, Germany; 4 Institute for Animal Breeding and Genetics, University of Veterinary Medicine Hannover (Foundation), 30559 Hanover, Germany; 5 Institute of Animal Breeding and Husbandry, Christian-Albrechts-University, 24118 Kiel, Germany; 6 Department of Animal Sciences, Georg-August-University, 37077 Göttingen, Germany; 7 Faculty of Agricultural and Environmental Sciences, University Rostock, 18059 Rostock, Germany

**Keywords:** Microsatellite, Single nucleotide polymorphism, Imputation, Equine parentage testing, Horse breeding

## Abstract

In horses, parentage control is currently performed based on an internationally standardized panel of 17 microsatellite (MS) markers comprising 12 mandatory and five optional markers. Unlike MS, single nucleotide polymorphism (SNP) profiles support a wider portfolio of genomic applications, including parentage control. A transition to SNP-based parentage control is favorable, but requires additional efforts for ensuring generation-overlapping availability of marker genotypes of the same type. To avoid double genotyping of either parents or offspring for changing to SNP technology and enable efficient transition, we tested whether MS genotypes used for parentage control could be reliably imputed from a medium-density SNP panel in German warmblood horses. Imputation accuracy was tested in a tenfold cross-validation with two approaches: within breed (option A) and across breeds (option B). Average imputation accuracies of 97.98% (A) and 96.17% (B) were achieved, respectively. Due to interbreed differences in genotyping rates, five MS markers of low genotyping rate (GTR; < 90%) could be imputed with higher accuracy within breed (98.18%) than across breeds (90.73%). MS markers with high GTR performed homogeneously well in option B (98.44%) and showed slightly lower accuracy in option A (97.90%). Among these markers, AHT5 proved to be problematic for imputation regardless of the approach, revealing accuracies of 86.40% (A) and 88.70% (B). Better results for MS markers with high GTR and savings in computational processing justified the choice of option B for routine implementation. To date, more than 9500 horses have undergone the new parentage control based on imputed MS genotypes.

**Supplementary Information:**

The online version contains supplementary material available at 10.1007/s13353-022-00725-9.

## Introduction

For more than two decades, parentage control for horses in Germany has been carried out using the microsatellite (MS) parentage panel recommended by the International Society for Animal Genetics (ISAG, https://www.isag.us/) (Bowling et al. 1997). The current MS panel includes a total of 17 MS, out of which 12 MS (AHT4, AHT5, ASB17, ASB2, ASB23, HMS2, HMS3, HMS6, HMS7, HTG10, HTG4, and VHL20) are mandatory for parentage control and five MS (CA425, HMS1, HTG6, HTG7, and LEX3) are optional. The internationally agreed standard of MS profiling facilitates horse trade and exchange of breeding stock. Horse breeding associations worldwide are aiming to introduce genomic selection, with several research initiatives in the sport horse sector. In horses, a high potential genetic gain via genomic selection is anticipated due to long generation intervals and because most economically important traits can only be measured relatively late in an animal’s life and show low heritabilities (Haberland et al. 2012; Stock et al. 2016). To promote application of genomic information in horse breeding, the International Association for Future Horse Breeding (IAFH) was founded in 2017 by the horse breeding associations of the Oldenburg (OL), the Oldenburg International (OS), the Westphalian (WESTF), the Trakehner (TRAK), and the Holsteiner (HOL) horse. As a prerequisite for genomic breeding value estimation, information on genome-wide single nucleotide polymorphisms (SNPs) is needed from the active breeding stock, foals, and subsequent generations of horses. Conditional on an appropriate number and selection of SNP markers, SNP genotypes can be used for estimating genomic breeding values for multiple traits (Meuwissen et al. 2016), for population monitoring and diversity management (Lee et al. 2014) as well as for parentage control (Holl et al. 2017). Compared to MS, SNPs have a lower mutation rate as reviewed by Vignal et al. (2002) and facilitate unequivocal standardization of alleles. At the same time, easier automation of the analytical process in the laboratory decreases costs and enables higher throughput in data generation for SNP genotyping (McClure et al. 2013; Vignal et al. 2002).

In parentage control, it is essential that parents and foals have the same type of information, i.e., either MS or SNP data must be available across generations. In order to avoid the effort and costs of genotyping horses twice over several generations during the transition phase from MS to SNPs, foals should be genotyped with only a SNP panel as early as possible.

A feasible transition between both categories of genotype information is the imputation of MS genotypes from SNP genotypes, which has already been successfully applied in other livestock species such as sheep (Marina et al. 2021) and cattle (McClure 2014; McClure et al. 2012). The development of SNP-based MS imputation for horses implies that new-born foals could be SNP genotyped and then receive imputed MS genotypes, which allows them to be matched with their MS genotyped sire and dam. Imputing foal MS genotypes also overcomes difficulties in the transition phase from MS to SNP genotyping, when parental samples for SNP genotyping are not available anymore.

Imputation uses SNP data to determine statistically, which MS genotypes an animal is most likely to carry. A reference panel or so-called training set is a basic requirement for a reliable imputation of MS genotypes. Ideally, the training set comprises several thousands of animals having been genotyped for both SNPs and MS. Furthermore, the reference panel should contain animals of the same breed or closely related breeds to the animals in the target panel. Before implementing a new procedure for parentage control in routine practice, it is crucially important to evaluate error rates and subsequently optimize the process. From other species, such as cattle, it is known that imputation of MS genotypes from SNP genotypes is possible, but the accuracy of imputation depends on the size of the reference panel and varies between within- and across-breed approaches (McClure et al. 2013, 2012). In our study, we aimed to determine the MS imputation accuracies from SNPs for different imputation strategies in German warmblood horses in order to select a suitable method for implementation into breeding practice. Here, we report the development, testing of different strategies, validation, and first experiences from implementation of SNP-based MS imputation in the German population of warmblood horses.

## Methods

### Testing and development

#### Dataset

The study cohort for testing and development comprised 2878 mares of the five warmblood horse breeds Oldenburg (OL, *N* = 958), Oldenburg International (OS, *N* = 179), Westphalian (WESTF, *N* = 249), Trakehner (TRAK, *N* = 430), and Holsteiner (HOL, *N* = 1062). Horses included in the study cohort were selected for unrelatedness to maximize genetic diversity in the reference population. To this end, we allowed a maximum of 20% kinship between two individuals. The horse genotype data was organized and processed in four batches: cohort 1, 2, 3, and 4.

Out of the 2878 horses, 2739 horses were genotyped for 12 to 17 MS, and 2809 horses were genotyped for at least 70 K genome wide SNPs using the GGP Equine 70 k or GGP Equine Plus genotyping beadchip (GeneSeek Genomic Profiler, Illumina). MS genotyping was carried out in different laboratories with varying standards, resulting in unequal genotyping rates (GTR) for the 17 MS (see Supplementary file 1). For MS, the ISAG nomenclature (https://strbase.nist.gov/horseSTRs.htm, accessed 21st November 2018) was used, which attributes alphabetic letters to MS alleles, depending on the repeat number (Van De Goor et al. 2010). For MS data, a VCF file was created manually in R (R Core Team 2018). In the VCF files, the MS alleles were coded as the repeat sequence times the respective repeat number. Alleles were separated by comma, starting with the lowest repeat number and ending with the highest repeat number. Genotypes were coded as the respective allele number from this comma-separated list.

The data per SNP and sample were filtered according to the following parameter settings: minor allele frequency (MAF) ≥ 0.01, GC score ≥ 0.6, call frequency ≥ 90%, call rate ≥ 95%, and Hardy–Weinberg equilibrium (HWE) *p* ≤ 0.001. After filtering and selecting for horses with information for both MS and SNP genotypes, a set of 2607 horses remained for further analyses, including 988 HOL, 926 OL, 164 OS, 333 TRAK, and 196 WESTF (see Supplementary file 2). Duplicate markers from the two SNP genotyping panels were removed prior to imputation, i.e., only one marker per position was kept in the dataset. A total of 60,197 SNPs from the initial two genotyping panels were kept after the merging and filtering steps. Next to manually creating a VCF file for MS genotype data, the dataset was merged with the SNP genotypes into a single VCF file. Restructuring and arranging of SNP and MS data was done with PLINK version 1.9 (Purcell et al. 2007) and vcftools version 0.1.16 (Danecek et al. 2011).

#### Model for analyses

MS allele frequencies were calculated per MS marker as the relative incidence of an allele at the given locus per breed as well as across breeds. The polymorphic information content (PIC) was calculated according to Botstein et al. (1980) for each of the 17 MS within the five breeds and across breeds. The calculations were done with the R-package polysat version 1.7 (Clark & Schreier 2017). Because Holsteiner horses were not genotyped for markers CA425, HMS1, and LEX3, the overall PIC was calculated across the other four breeds for these markers. For each of the MS markers, the observed heterozygosity (*H*_*O*_) was calculated separately per breed and for all animals together.

Imputation of MS genotypes and missing SNP genotypes was carried out in a one-step approach using BEAGLE 5.0 (Browning et al. 2018). To enhance accuracy, imputation parameters were set to a window size of 200 and an effective population size of 30,000 according to initial settings made by Pook (2019) and Pook et al. (2020). All other parameters were kept at default values.

To test MS allele imputation accuracies, a tenfold cross-validation with the following study design was used. We randomly selected 10% of all animals (*N* = 261), proportionally distributed among the five breeds (99 HOL, 93 OLD, 16 OS, 20 WESTF, 33 TRAK), masked their MS genotypes and subsequently imputed these MS genotypes based on their SNP genotypes and the SNP and MS genotypes of the other 90% of the training set. This procedure was repeated in ten replicates (test rounds). We allowed animals to be included in more than one of the ten randomly selected and masked replicates. Per test round, two different scenarios were tested for average imputation accuracies: (A) within breed, i.e., considering animals from the same breed only, and (B) across breeds, i.e., considering all animals from all five breeds together. The imputed MS alleles were then compared to the true MS alleles of the validation animals. For accuracy testing, a score was assigned for each animal and each microsatellite: 0 (both alleles incorrectly imputed, i.e., differing from the original), 0.5 (one allele correctly imputed), and 1 (both alleles correctly imputed). Average accuracy scores per replicate, marker, individual, and breed were then determined as the arithmetic mean.

### Validation and implementation

With the beginning of the breeding season 2021, four of the five studbooks of the IAFH (OL, OS, HOL, and TRAK) have started with routine SNP genotyping and parentage control via MS imputation from SNPs for all foals born. For this practical application, the computation center and IT service provider of the studbooks, vit (Verden, Germany) adopted the developed and validated system described above. The vit integrated this new approach into a routine process, which was rigorously tested in summer 2021.

Sampling of horses (mostly foals) requiring parentage control for registration was organized by the breeding organizations. Sample processing, DNA isolation, and SNP genotyping were performed in the molecular genetic laboratory (IFN Schönow GmbH, Schönow, Germany) following standard protocols and using the Equine80select genotyping beadchip (Illumina). Beagle software (version 5.1) was used for imputing with imputation parameters analogous to the initial testing and development stage, except for effective population size, which was lowered to 3000.

For extended validation, all horses with available SNP and MS genotypes by July 12, 2021 (*N* = 5138) were used and analyzed in different scenarios. The validation dataset included different warmblood breeds and few thoroughbred horses used in warmblood breeding. Horses had been genotyped with at least medium-density SNP arrays between 2017 and 2021 and had MS genotypes for at least 10 of 12 MS markers from the currently recommended ISAG panel of MS (Supplementary file 2).

To investigate possible options for optimizing overall performance of the imputation routine in terms of run time and imputation accuracy, the number of SNPs around the MS was modified by defining four different window sizes: the whole chromosome (on which the MS is located), 5, 3.5, or 2 Mb up- and downstream of each MS. In addition, different ratios between the number of validation animals and the number of reference animals were used: the number of validation animals was set at 10%, 25%, 65%, or 150% in relation to the number of reference animals, corresponding to absolute numbers *N* = 500, *N* = 1000, *N* = 2000, and *N* = 3000 validation animals, respectively. Combining these settings resulted in a total of 16 validation scenarios. Each of these scenarios was repeated ten times with randomly chosen validation animals, for which the MS genotypes were masked and subsequently imputed. The imputation accuracy was calculated for each MS and averaged across all replicates of each scenario.

Finally, the new MS imputation was implemented in the routine parentage control pipeline of horses for all 12 core panel MS markers and two optional markers (HTG6, HTG7). To fully exploit all SNP genotype information, the option of including all SNPs located on chromosomes with MS genotype information was selected. Subsequent parentage testing was performed by comparing the imputed MS genotypes of offspring with the MS genotypes of their parents, which were available through the electronic studbooks of the breeding organizations. Because of the poor imputation accuracy for AHT5, it was decided not to include this MS in the practical routine. Indications of Mendelian conflicts were verified by MS genotyping in the same laboratory and using the same DNA samples as for SNP genotyping. In case of suspected sampling errors, re-sampling of respective horses was organized by the studbooks, followed by MS genotyping for repeated parentage testing. In all horses, for which MS genotypes were available from the new imputing pipeline and also from additional laboratory genotyping analyses, the consistency of data was checked between the two data sources. Allelic differences were counted separately for MS included in the current ISAG core panel and the optional MS to reflect the definition of penalized Mendelian conflicts.

## Results

### Testing and development

#### Microsatellite genotyping rates, allele frequencies, and polymorphic information content

Due to different studbook and laboratory standards, we observed strong differences in GTRs of MS (see Supplementary file 1). The GTR for MS ranged between 1.86% (LEX3) and 100% across all breeds (see Supplementary file 1). Subsequently, MS with an average GTR above or below 90% will be referred to as high GTR and low GTR, respectively. Genotypes for ASB17 (61.64%) and ASB23 (61.53%) were mostly missing for one breed (HOL). MS markers with low GTR were ASB17, ASB23, CA425, HMS1, and LEX3. For some MS, e.g., HMS2 and VHL20, we observed large differences in allele frequencies between breeds, up to breed-specific major alleles (Supplementary file 1).

#### Imputation accuracy overall and per scenario

**Fig. 1:**
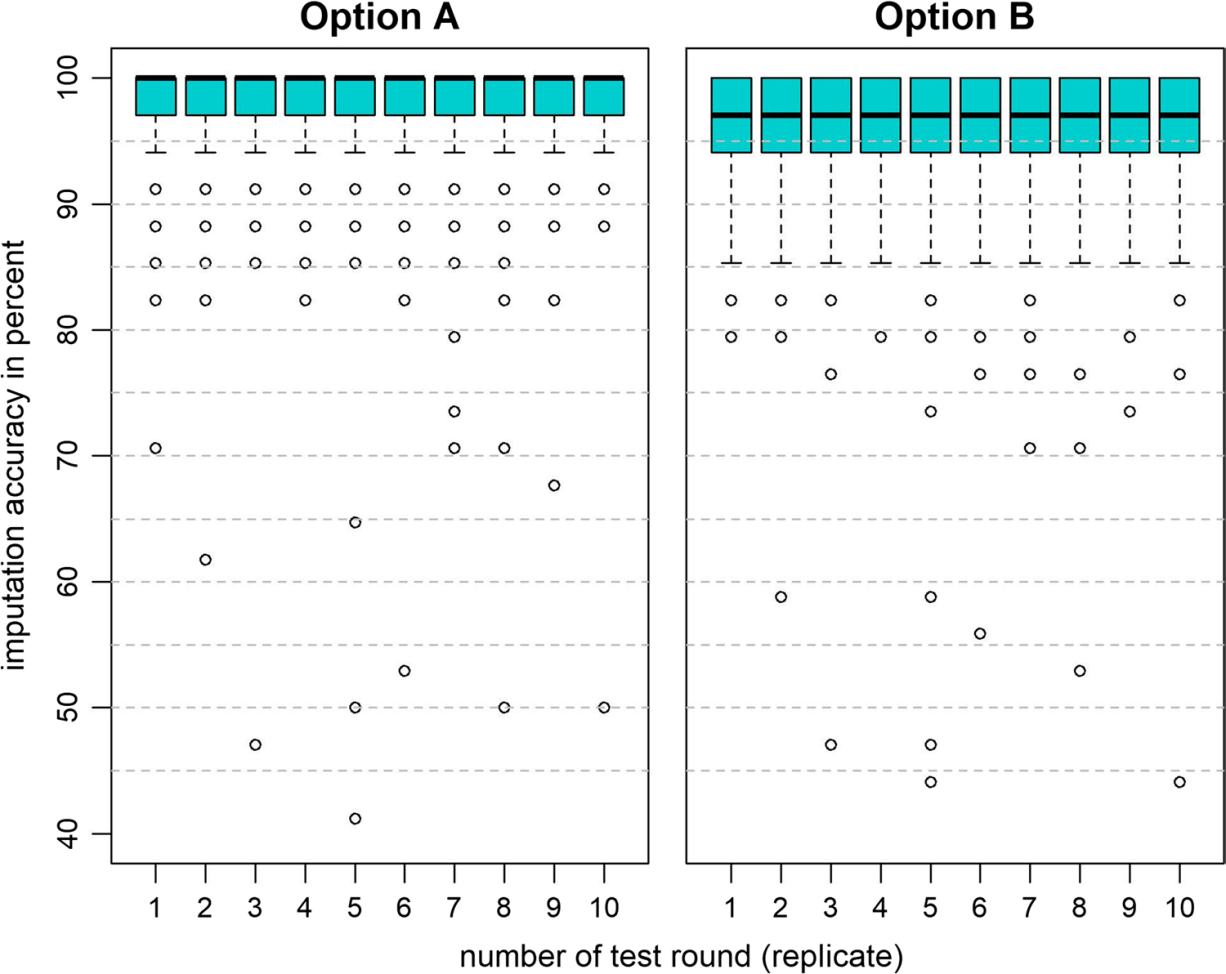
Distribution of imputation accuracies of 17 ISAG-panel microsatellite markers for the validation animals (*N* = 261) in ten replicates in option A (within breeds) and option B (across breeds)

The final SNP dataset, comprising 60,197 SNPs after filtering, had an average GTR of 98.85% (± 0.49%). The average MAF was 0.27 (± 0.14) (Supplementary file 3). Considering all results per animal across the ten replicates, mean imputation accuracies of 97.98% ± 4.02 (median: 100%) and 96.17% ± 4.68 (median: 97.06%) were obtained in options A and B, respectively (see Supplementary file 4). The mean accuracies per replicate ranged from 97.59 to 98.28% in option A and from 95.97 to 96.35% in option B. Single outlier animals scored as low as 41.18% in option A and 44.12% in option B (see Fig. 1). Thereby, results from option B were slightly lower than in option A. Generally, the results per replicate were very homogenous within each option.

**Fig. 2:**
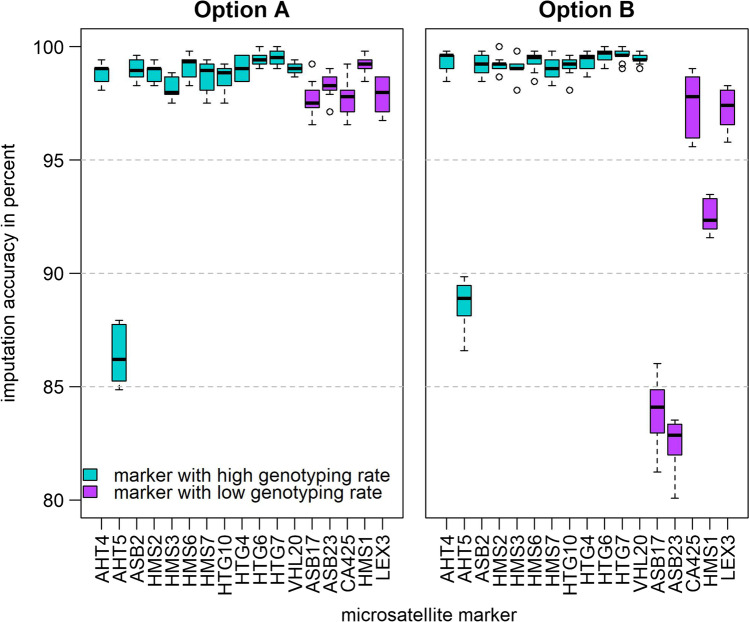
Distribution of imputation accuracies at microsatellite marker level across ten replicates in option A (within breeds) and option B (across breeds). High genotyping rate (GTR) markers are colored in turquoise and low GTR markers in purple. Low GTR markers are characterized by a lower imputation accuracy compared with high GTR markers in both imputation scenarios

The mean imputation accuracies for low GTR MS markers (ASB17, ASB23, CA425, HMS1, LEX3) were clearly higher in a within-breed approach (option A, mean: 98.18%, SD: 0.58%) than in an across-breeds approach (option B, mean: 90.73%, SD: 6.47%) (Fig. 2 and Supplementary file 4). In contrast, high GTR MS markers benefited from an across-breeds approach (option B, mean: 98.44%, SD: 2.94%) compared with the within-breed approach (option A, mean: 97.90%, SD: 3.47%). Out of all high GTR MS markers, AHT5 proved to be the most problematic one, while performing clearly better in option B (mean: 88.70%, SD: 1.09%) than in option A (mean: 86.40%, SD: 1.20%). With regard to the high GTR markers, HTG6 and HTG7 performed best both in option A (HTG6 mean: 99.43%, HTG7 mean: 99.52%) and B (HTG6 mean: 99.62%, HTG7 mean: 99.62%).

**Fig. 3:**
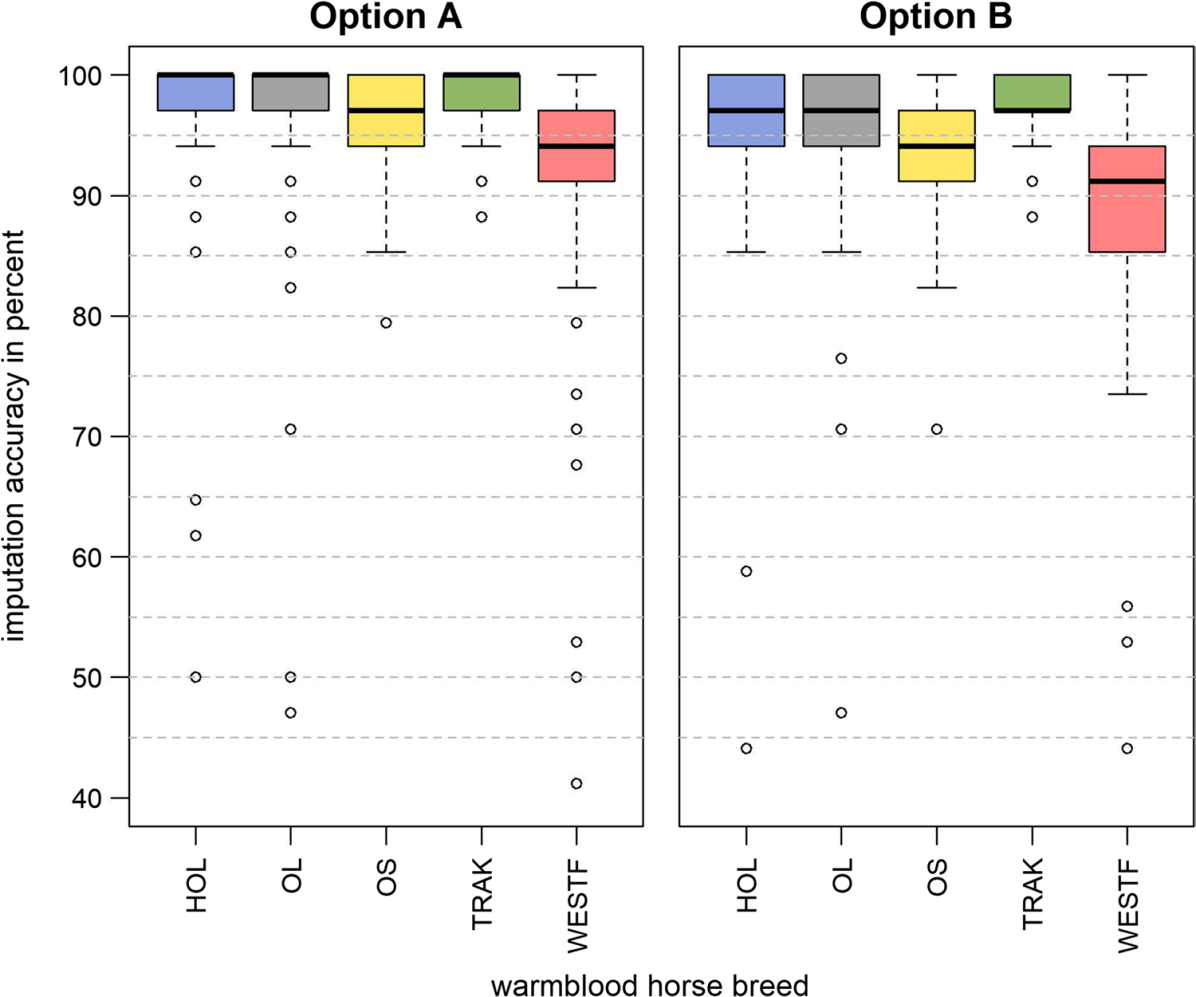
Distribution of imputation accuracies of 17 ISAG-panel microsatellite markers for five warmblood horse breeds across ten replicates in option A (within breeds) and option B (across breeds)

Considering the five breeds individually, all of them performed better in option A when all 17 MS markers were involved (Fig. 3). In option A, HOL achieved the best results (mean: 98.76%, SD: 2.98%), followed by TRAK (mean: 98.41%, SD: 2.26%), OL (mean: 98.25%, SD: 3.62%), OS (mean: 96.69%, SD: 3.52%), and, scoring comparatively low, WESTF (mean: 93.16%, SD: 7.75). Differences observed between the two options A and B were due to achieved imputation accuracies for those markers that differed in GTR. When only those 12 high GTR MS markers were considered, for which MS genotype data were available for almost all horses, the results between both options harmonized and option B rendered higher imputation accuracies with 97.81% and above (Supplementary file 5).

### Validation and implementation

#### Imputation accuracy per scenario

In the different scenarios with window sizes comprising 2.0, 3.5, and 5.0 Mb up- and downstream of each MS, the numbers of SNPs used for imputing varied widely, with substantial effect of the chromosomal location on the number of available SNPs (Supplementary file 6). The mean imputation accuracies ranged from 95.0 to 99.6% across all scenarios for 13 of the 14 MS included in the new imputation routine (AHT4, ASB2, ASB17, ASB23, HMS2, HMS3, HMS6, HMS7, HTG10, HTG4, VHL20 from the ISAG core panel, and HTG6 and HTG7 as ISAG additional MS). The mean accuracy for AHT5 was substantially lower and ranged from 83.3 to 91.6% (Table 1).

**Table 1:** Mean imputation accuracies of the ten validation runs for each imputed MS and each of the 16 scenarios defined by SNP window sizes around the MS and the ratio between the number of samples in the validation and reference set (10%, 25%, 65%, or 150%)

	VAL/REF^1^ 10%				VAL/REF 25%				VAL/REF 65%				VAL/REF 150%			
Mb^2^	2	3.5	5	all^3^	2	3.5	5	all	2	3.5	5	all	2	3.5	5	all
MS																
AHT4	99.3	99.3	99.3	99.2	99.1	99.1	99.1	99.0	99.0	99.0	99.0	98.9	98.9	98.8	98.8	98.8
AHT5	84.2	86.4	89.4	91.6	83.8	85.8	88.6	91.2	83.7	85.9	88.4	90.9	83.3	85.2	87.7	89.7
ASB2	99.1	99.1	99.0	99.1	99.2	99.2	99.2	99.2	99.0	99.0	99.0	99.0	98.9	98.8	98.8	98.9
ASB17	97.3	95.8	97.4	98.5	97.0	95.8	96.9	98.0	96.5	95.3	97.3	98.2	96.5	95.0	96.4	97.9
ASB23	98.5	98.2	98.2	98.7	97.7	97.8	97.4	98.4	98.2	97.7	97.8	98.4	98.1	97.9	97.5	98.2
HMS2	99.2	99.1	99.2	99.1	99.1	99.1	99.1	99.1	99.0	99.0	99.0	99.0	98.8	98.8	98.8	98.8
HMS3	98.9	98.9	98.9	98.8	98.8	98.8	98.8	98.8	98.8	98.8	98.8	98.8	98.6	98.6	98.6	98.6
HMS6	99.2	99.2	99.1	99.1	99.3	99.3	99.2	99.1	99.2	99.2	99.2	99.1	99.1	99.1	99.0	98.9
HMS7	99.1	99.1	99.1	98.9	99.1	99.1	99.0	99.0	99.0	99.0	99.0	98.9	98.9	98.9	98.9	98.8
HTG10	99.3	99.4	99.4	99.3	99.3	99.3	99.3	99.2	99.3	99.3	99.3	99.2	99.1	99.1	99.1	99.0
HTG4	99.4	99.4	99.4	99.4	99.3	99.3	99.3	99.2	99.4	99.4	99.4	99.3	99.2	99.2	99.2	99.2
HTG6	99.5	99.6	99.6	99.5	99.6	99.5	99.5	99.5	99.5	99.4	99.4	99.4	99.4	99.4	99.4	99.4
HTG7	99.3	99.3	99.3	99.5	99.2	99.3	99.2	99.4	99.2	99.3	99.3	99.4	99.1	99.2	99.1	99.4
VHL20	99.0	99.1	99.0	99.0	99.0	99.1	99.0	99.0	99.0	99.0	99.0	99.0	98.8	98.9	98.8	98.8

^1^VAL/REF = ratio of validation animals to reference animals in percent, ^2^ Mb = megabase pairs considered around the MS (2, 3.5, or 5 Mb up- and downstream), ^3^all = entire chromosome where the MS is located was considered

**Fig. 4:**
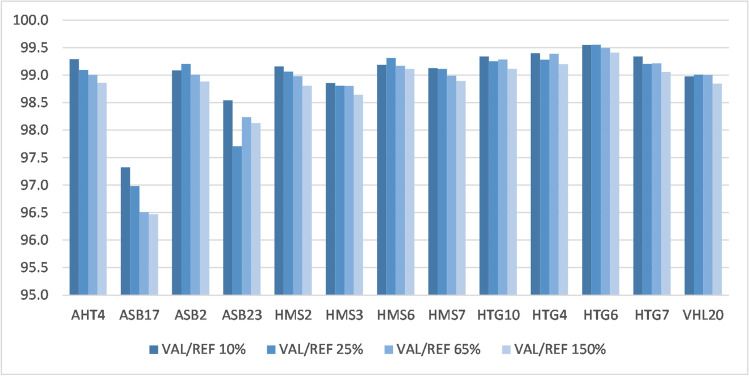
MS imputation accuracies for different ratios between the numbers of animals in the validation set and in the reference set, considering all SNPs within a window size of 2 Mb around the MS on the respective chromosome

**Fig. 5:**
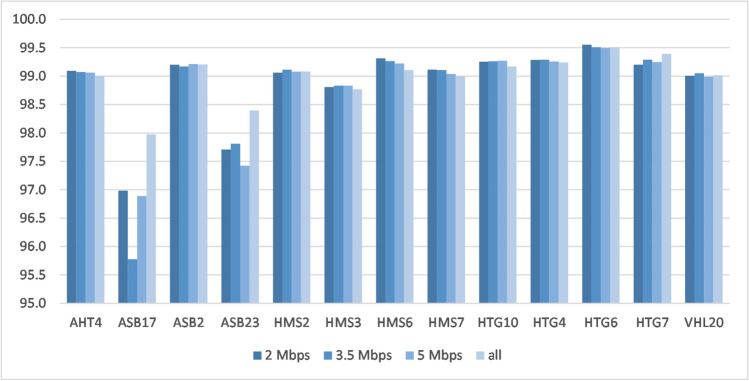
MS imputation accuracies for different window sizes around the MS on the respective chromosome with a ratio of validation to reference animals of 25% (*N* = 1000 validation animals)

Imputation accuracies improved with increasing numbers of animals in the reference set, i.e., when the ratio of validation animals to reference animals was lower. For MS with lower GTR within the reference set (ASB17 and ASB23), the accuracies increased when more SNPs around the MS were used for the imputation process (Figs. 4 and 5).

For graphical illustration of the mean accuracies of all 16 validation scenarios for each of the 14 imputed MS markers, see Supplementary file 6.

#### Mendelian errors and allelic differences

Until October 18, 2021, *N* = 9590 samples of horses from the four aforementioned studbooks have passed the imputation procedure in the parentage control routine. Experimental MS genotyping due to indications of Mendelian conflicts was required for 1.4% of the samples (*N* = 130) with minor differences between the studbooks (Table 2). Among those 115 samples, for which experimental MS genotyping results became available for verification, Mendelian conflicts were confirmed in 81% of the cases.

**Table 2:** Distribution of horse samples under parentage control based on MS imputation across studbooks, with specification of the number and proportion of samples with indications of Mendelian conflicts, which were subsequently verified by experimental laboratory MS genotyping

	TRAK	HOL	OL	OS	Total
Number of samples without indications of Mendelian conflicts	982	2383	3363	2718	9446
Number of samples not allowing SNP genotyping	6	1	3	4	14
Number (%) of samples with indications of Mendelian conflicts	12 (1.2%)	25 (1.0%)	54 (1.6%)	39 (1.4%)	130 (1.4%)
Number (%) of samples confirmed Mendelian conflicts	9 of 10 (90%)	13 of 16 (81%)	46 of 54 (85%)	25 of 35 (71%)	93 of 115 (81%)
Total number of horses	1000	2409	3420	2761	9590

In addition to the 115 samples with putative Mendelian errors, MS genotypes from both routine MS imputing and laboratory analysis became available for 93 further samples. Of these 208 horses in total, a maximum of 89.4% had a discordant MS allele across all 13 routinely reported MS markers. The proportion of horses with two allelic differences was 8.6%, and a further 1.4% of the horses had three allele divergences. In a single case, imputation revealed differences for 12 of the 13 MS markers, with no correctly imputed MS alleles for one marker (HMS6) and only one correctly imputed MS alleles for 11 markers (Supplementary file 2).

## Discussion

In this study, we aimed to establish a protocol for imputing microsatellite (MS) genotypes based on SNP genotypes in order to provide imputed MS genotypes for parentage control. The dataset used in this study comprised 2607 mares from the five German warmblood horse breeds Holsteiner (HOL), Oldenburger (OL), Oldenburg International (OS), Trakehner (TRAK), and Westphalian (WESTF). All animals were genotyped with at least 70 K SNPs using a commercially available medium-density SNP beadchip and were also genotyped for a set consisting of 12 to 17 MS markers. MS GTRs were 99.93% across 12 high GTR MS markers and 26.92% across further five low GTR MS markers. Breed specific GTRs for low GTR MS markers varied between 0 and 99.49%, depending on studbook and lab specific standards.

Female horses were chosen for SNP genotyping, because sampling could be organized in connection with comprehensive phenotyping at routinely executed performance tests and studbook registrations. When compared to stallions, female horses are presented to the studbooks in larger numbers and with a lower degree of preselection implying their advantageous use to build-up a reference population for genomic evaluation. This study has also likely benefitted from the higher diversity of a mainly female training set for SNP-based MS imputation.

Although breed-specific MS alleles were observed in our dataset, these had very low allele frequencies. For the MS markers AHT4 and HMS6, all breeds had the same major alleles (O and P, respectively) and for the MS markers AHT5, HTG4, HTG6, HTG7, and VHL20, four out of five breeds had the same major alleles. A high similarity of breeds at a MS level could therefore be assumed. Combined with the rather strong connectedness between the breeds (Nolte et al. 2019; Vosgerau et al. 2022), an imputation of all breeds together seemed feasible.

In a tenfold cross-validation, we tested whether MS genotypes could accurately be imputed based on SNP genotypes in warmblood horses. In each of the ten test replicates, the MS genotypes of 10% of the animals were masked and then imputed based on the other 90% of the animals.

Initially, imputation accuracy appeared to be better in a breed-specific approach (option A), but further investigation showed that this was mostly due to low GTR MS markers. Markers with low GTR performed quite differently in the within-breed approach (option A, mean imputation accuracy of low GTR markers: 98.18%) compared with the across-breeds approach (option B, mean imputation accuracy of low GTR markers: 90.73%). However, this advantage of option A disappeared when only high GTR markers were considered. Here, the across-breeds approach (option B, mean imputation accuracy of high GTR markers: 98.44%) outperformed the within-breed approach by 0.54%. The uneven distribution of the data for the low GTR MS markers across breeds, with very low GTRs in some breeds, was obviously problematic for MS imputation in option B. However, high GTR markers have benefitted from an across-breeds approach because a much higher number of animals were in the training subset. We conclude that a high quantity of information leads to better imputation accuracies.

Compared to previous results in cattle (McClure 2014), a similar imputation accuracy was achieved within our datasets, particularly when considering MS markers with GTRs above 90%. This is remarkable, especially because the reference panel in each replicate contained less than 2500 animals, while McClure (2014) used a reference panel of over 7000 animals and reached accuracy levels similar to our study (above 98%) in Irish cattle. With over 97% imputation accuracy, comparable magnitudes have also been reported from Spanish Assaf sheep, following an experimental design (Marina et al. 2021), which was almost identical to the design in our study. However, expanding our imputation scheme to other warmblood breeds or even pony or draft horse breeds, which are more distantly related, ought to be done with caution and applied only after additional validation. From studies in cattle, Přibáňová et al. (2020) and Sharma et al. (2018) have shown that discordances arise more frequently in animals that belong to breeds that are less closely related to the reference panel. These findings are in agreement with our experience from the first breeding season, in which imputed MS were used for routine parentage testing. To avoid erroneous questioning of pedigrees, indications of Mendelian conflicts arising from the comparison of imputed MS genotypes of the progeny with parental MS genotypes had to be confirmed experimentally by MS genotyping of the progeny in the laboratory. The confirmation rate of only 81% among 208 checked samples and the distribution of the 22 non-confirmed Mendelian conflicts reflected the impact of quantity and quality of information on MS allele distributions on imputation accuracies. The low GTR marker ASB17 was among the MS markers with most discordances, and the affected pedigrees mostly contained horses from breeds which were not or only sparsely represented in the training set at least for some of the MS (non-German warmblood breeds, Holstein).

High imputation accuracy in our study might not only be due to the population structure and relatedness between validation and reference subsets of replicates, but also because of the comparatively high SNP density and SNP call rate of over 95%. Přibáňová et al. (2020) concluded from imputation results in cattle that a SNP call rate of over 90% is necessary to build reliable haplotypes, especially when SNPs are in close proximity to the MS. In the routine setting, the average SNP call rate was even higher than in the development study (> 98%), implying very good conditions for a high MS imputation accuracies. In the single case, where multiple discordances were observed between imputed MS genotypes and MS genotypes generated in the laboratory, the initially implemented filtering practice using primarily the GC score proved to be insufficient: despite a SNP call rate of only 80%, multiple SNPs had passed the threshold of GC score > 0.6. The poor imputation accuracies across all MS for this sample underline the importance of a SNP call rate > 90% as an additional filter criterion for SNP genotype data to be used for MS imputation.

The recommendations for parameter settings in Beagle regarding window size and effective population size (Ne) made by Pook (2019) have been concluded from studies in maize. Especially with regard to Ne, the recommended 30,000 are still strongly above estimations for horse populations, where a few hundred are more realistic (Corbin et al. 2010). This parameter has therefore been lowered to 3000 in the routine implementation.

Out of all high GTR markers, AHT5 very clearly presented the greatest challenge and repeatedly had the lowest accuracy scores. While this marker performed slightly better in option B (mean: 88.70%) compared with option A (mean: 86.40%), nevertheless both options yielded an insufficient accuracy level of less than 90%. Further investigation showed that this MS is located on a telomeric end of chromosome 8 at 0.74 Mb. Therefore, upstream of this MS, there are only eight SNPs located, which can be used for imputation. This clearly hampered high-quality results. In previous ISAG horse parentage comparison tests, AHT5 has also been recognized for a number of discordant results indicating problems with correct genotyping of this MS marker (ISAG 2019). Due to its telomeric and thereby problematic position and the obvious implications for imputation, we suggest to exclude AHT5 from parentage testing in horses, when imputed MS genotypes are used. For routine implementation, imputing results for this MS were ignored.

McClure et al. (2012) and Přibáňová et al. (2020) suggested setting up an automated pipeline that identifies animals with rare haplotypes and failed parentage verification due to the imputed MS. By re-genotyping these animals for MS markers and adding them to the reference population, the imputation accuracy can subsequently be improved. This concept has already been implemented in our imputation strategy when discordances between pedigree and imputed MS genotypes occur (not considering AHT5).

In summary, we presented an imputation approach in warmblood horses that reached very high accuracies, which is a prerequisite for parentage verification based on imputed MS genotypes. Double genotyping of new-born foals of the major German horse breeds Holsteiner, Oldenburger, Oldenburg International, and Trakehner will therefore not be necessary. A final statement on whether the method for imputing MS genotypes also provides accurate results for Westphalian horses can only be made as soon as horses from this studbook have passed through the routine in sufficient numbers. Consequently, the MS genotype imputation results in a drastic cost reduction for breeders and studbooks. Our results can be considered very encouraging for the future. By including more horses for each of the breeds, it should be possible to further increase the accuracies. Additional studies are needed to test if MS imputation in other, more distantly related equine breeds, including pony or draft horse breeds, could also be performed based on this dataset. Similar investigations are recommended for stallions of foreign breeds with a genetic contribution to the German warmblood horse population.

## Supplementary Information

Below is the link to the electronic supplementary material.

Supplementary file1 (XLSX 41 KB)

Supplementary file2 (XLSX 21 KB)

Supplementary file3 (CSV 2822 KB)

Supplementary file4 (XLSX 23 KB)

Supplementary file5 (PDF 240 KB)

Supplementary file6 (XLSX 144 KB)

## Data Availability

Genotyping data is available upon reasonable request.

## References

[CR1] Botstein D, White RL, Skolnick M, Davis RW (1980). Construction of a genetic linkage map in man using restriction fragment length polymorphisms. Am J Hum Genet.

[CR2] Bowling AT, Eggleston-Stott ML, Byrns G, Clark RS, Dileanis S, Wictum E (1997). Validation of microsatellite markers for routine horse parentage testing. Anim Genet.

[CR3] Browning BL, Zhou Y, Browning SR (2018). A one-penny imputed genome from next-generation reference panels. Am J Hum Genet.

[CR4] Clark LV, Schreier AD (2017). Resolving microsatellite genotype ambiguity in populations of allopolyploid and diploidized autopolyploid organisms using negative correlations between allelic variables. Mol Ecol Resour.

[CR5] Corbin LJ, Blott SC, Swinburne JE, Vaudin M, Bishop SC, Woolliams JA (2010). Linkage disequilibrium and historical effective population size in the thoroughbred horse. Anim Genet.

[CR6] Danecek P, Auton A, Abecasis G, Albers CA, Banks E, DePristo MA, Handsaker RE, Lunter G, Marth GT, Sherry ST, McVean G, Durbin R (2011). The variant call format and VCFtools. Bioinformatics.

[CR7] Haberland AM, König von Borstel U, Simianer H, König S (2012). Integration of genomic information into sport horse breeding programs for optimization of accuracy of selection. Animal.

[CR8] Holl HM, Vanhnasy J, Everts RE, Hoefs-Martin K, Cook D, Brooks SA, Carpenter ML, Bustamante CD, Lafayette C (2017). Single nucleotide polymorphisms for DNA typing in the domestic horse. Anim Genet.

[CR9] ISAG (2019) Equine genetics and thoroughbred parentage testing (workshop report). Lleida (Spain).

[CR10] Lee Y-S, Woo Lee J, Kim H (2014). Estimating effective population size of thoroughbred horses using linkage disequilibrium and theta (4Nμ) value. Livest Sci.

[CR11] Marina H, Suarez-Vega A, Pelayo R, Gutiérrez-Gil B, Reverter A, Esteban-Blanco C, Arranz JJ (2021). Accuracy of imputation of microsatellite markers from a 50K SNP chip in Spanish Assaf sheep. Animals (basel).

[CR12] McClure M, Sonstegard T, Wiggans G, Van Tassell C (2012). Imputation of microsatellite alleles from dense SNP genotypes for parental verification. Front Genet.

[CR13] McClure M, Sonstegard T, Wiggans G, Van Eenennaam A, Weber K, Penedo MC, Berry D, Flynn J, Garcia JF, Carmo A, Regitano L, Albuquerque M, Silva M, Machado M, Coffey M, Moore K, Boscher M-Y, Genestout L, Mazza R, Taylor J, Schnabel R, Simpson B, Marques E, McEwan J, Cromie A, Coutinho L, Kuehn L, Keele J, Piper E, Cook J, Williams R, Van Tassell C (2013). Imputation of microsatellite alleles from dense SNP genotypes for parentage verification across multiple Bos taurus and Bos indicus breeds. Front Genet.

[CR14] McClure M, Treacy, M, O’Connor, K, McCarthy, J, Kearney, J.F (2014) Accelerating the shift to SNP-based parentage verification through microsatellite imputation in Ireland. In: *World congress on genetics applied to livestock production*, p. 655, Vancouver (Canada).

[CR15] Meuwissen T, Hayes B, Goddard M (2016). Genomic selection: a paradigm shift in animal breeding. Anim Front.

[CR16] Nolte W, Thaller G, Kuehn C (2019). Selection signatures in four German warmblood horse breeds: tracing breeding history in the modern sport horse. PLoS ONE.

[CR17] Pook T (2019). Methods and software to enhance statistical analysis in large scale problems in breeding and quantitative genetics.

[CR18] Pook T, Mayer M, Geibel J, Weigend S, Cavero D, Schoen CC, Simianer H (2020) Improving imputation quality in BEAGLE for crop and livestock data. G3: Genes|Genomes|Genetics 10: 177.10.1534/g3.119.400798 10.1534/g3.119.400798 PMC6945036 31676508

[CR19] Přibáňová M, Schroffelová D, Lipovský D, Kučera J, Šteiger V, Hromádková J, Němcová L (2020). Use of SNPs from Illumina BovineSNP50K BeadChip v3 for imputation of microsatellite alleles for parentage verification and QTL reporting. Czech J Anim Sci.

[CR20] Purcell S, Neale B, Todd-Brown K, Thomas L, Ferreira MAR, Bender D, Maller J, Sklar P, de Bakker PIW, Daly MJ, Sham PC (2007). PLINK: a tool set for whole-genome association and population-based linkage analyses. Am J Hum Genet.

[CR21] R Core Team (2018) R: a language and environment for statistical computing. URL https://www.R-project.org/.

[CR22] Sharma A, Park JE, Park B, Park MN, Roh SH, Jung WY, Lee SH, Chai HH, Chang GW, Cho YM, Lim D (2018). Accuracy of imputation of microsatellite markers from BovineSNP50 and BovineHD BeadChip in Hanwoo population of Korea. Genomics & Informatics.

[CR23] Stock KF, Jönsson L, Ricard A, Mark T (2016). Genomic applications in horse breeding. Anim Front.

[CR24] Van De Goor LHP, Panneman H, Van Haeringen WA (2010). A proposal for standardization in forensic equine DNA typing: allele nomenclature for 17 equine-specific STR loci. Anim Genet.

[CR25] Vignal A, Milan D, SanCristobal M, Eggen A (2002). A review on SNP and other types of molecular markers and their use in animal genetics. Genet Sel Evol.

[CR26] Vosgerau S, Krattenmacher N, Falker-Gieske C, Seidel A, Tetens J, Stock KF, Nolte W, Wobbe M, Blaj I, Reents R, Kuhn C, Prondzinski MV, Kalm E, Thaller G (2022). Genetic and genomic characterization followed by single-step genomic evaluation of withers height in German warmblood horses. J Appl Genet.

