# Supplementary material for: Replacement of microsatellite markers by imputed medium-density SNP arrays for parentage control in German warmblood horses
Source: J Appl Genet. 2022 Sep 29;63(4):783–92. doi: 10.1007/s13353-022-00725-9 (PMC9637052; doi:10.1007/s13353-022-00725-9)

Supplementary file 5: Distribution of imputation accuracies of 12 high genotyping rate (GTR) microsatellite markers for five warmblood horse breeds across ten replicates in Option A (within breeds) and Option B (across breeds).

Replacement of microsatellite markers by imputed medium-density SNP arrays for parentage control in German warmblood horses

J Appl Genet

Wietje Nolte, Hatem Alkhoder, Mirell Wobbe, Kathrin F. Stock, Ernst Kalm, Sarah Vosgerau, Nina Krattenmacher, Georg Thaller, Jens Tetens and Christa Kühn
kuehn@fbn-dummerstorf.de

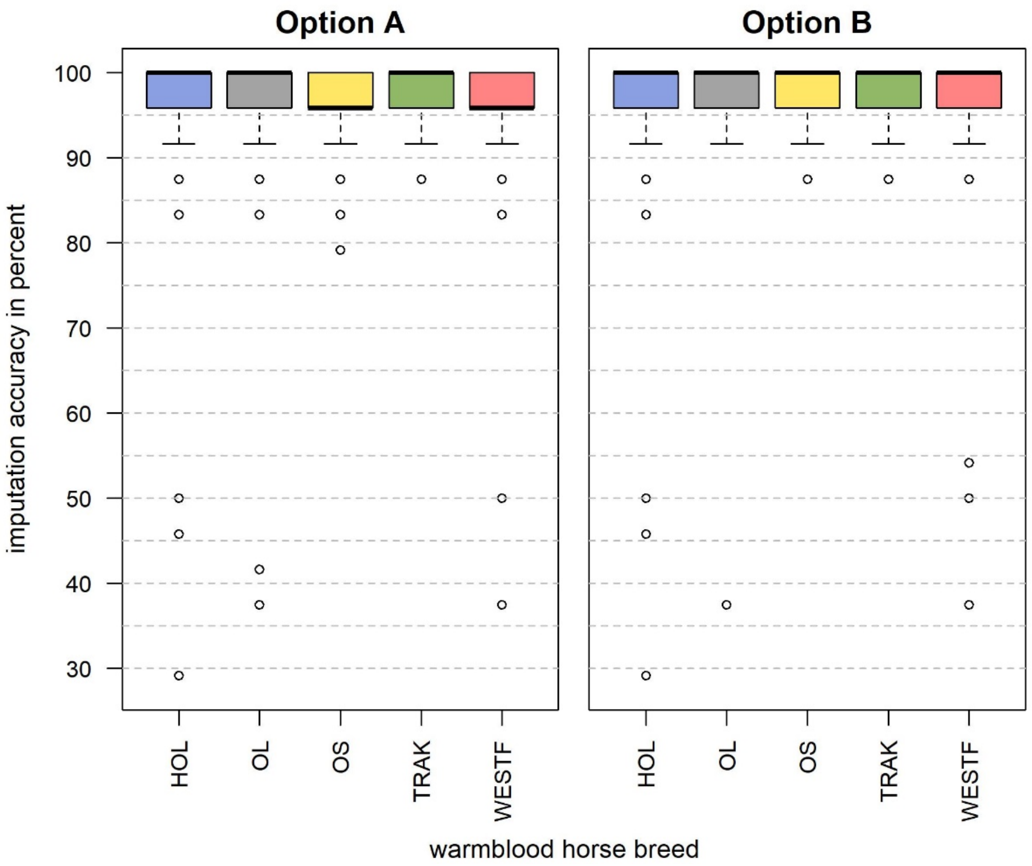

Supplement: Supplementary file 5 — Supplementary file5 (PDF 240 KB) [file 13353_2022_725_MOESM5_ESM.pdf]
